# The funding effectiveness in chemistry at the National Natural Science Foundation of China: research progress on the fundamentals and applications of zeolite materials

**DOI:** 10.1093/nsr/nwaf556

**Published:** 2025-12-05

**Authors:** Wei-Li Dai, Li-Hua Chen, Chang Yao, Bao-Lian Su, Jun-Lin Yang, Fei-Xue Gao

**Affiliations:** Department of Chemical Sciences, National Natural Science Foundation of China, Beijing 100085, China; Institute of New Catalytic Materials Science, School of Materials Science and Engineering, Nankai University, Tianjin 300350, China; School of Chemistry, Chemical Engineering and Life Sciences, State Key Laboratory of Advanced Technology for Materials Synthesis and Processing, Wuhan University of Technology, Wuhan 430070, China; Department of Chemical Sciences, National Natural Science Foundation of China, Beijing 100085, China; School of Chemistry, Chemical Engineering and Life Sciences, State Key Laboratory of Advanced Technology for Materials Synthesis and Processing, Wuhan University of Technology, Wuhan 430070, China; Department of Chemical Sciences, National Natural Science Foundation of China, Beijing 100085, China; Department of Chemical Sciences, National Natural Science Foundation of China, Beijing 100085, China

**Keywords:** science funding, funding effectiveness, zeolite, catalysis, adsorption and diffusion

## Abstract

Zeolites have undergone a significant development, leading to their widespread application in catalytic processes. China plays a pivotal role in the rapid growth of pioneering zeolite research. The National Natural Science Foundation of China (NSFC) has facilitated the transition of Chinese scientists from a position of research followers to a leading role on the global stage. The future development of zeolite science and technology in China depends on the sustained provision of innovations, advanced techniques and effective scientific funding from the Chinese government, including the NSFC. This paper firstly provides a comprehensive review of the funding projects in the field of zeolite research from the NSFC. Subsequently, a spotlight on new zeolite synthesis, novel characterization techniques and zeolite applications is presented to reveal the significance and effectiveness of these fundings. Finally, the future prospects of the NSFC supports in zeolites are envisaged.

## Teaser

An overview of funding projects related to zeolite in chemistry at the National Natural Science Foundation of China and a spotlight on important progress in this field.

## INTRODUCTION

Zeolites are inorganic crystalline microporous solids with frameworks composed of corner-sharing tetrahedral TO_4_ units (T = Si, Al, P, etc.), forming periodic 1D to 3D channels with a typical aperture size of <2 nm. Due to their unique pore structure, large specific surface area, suitable acidity, high thermal/hydrothermal stability, shape selectivity and molecular-recognition effect, zeolites are widely used as catalysts, adsorbates and ion exchangers in the oil-refining, petrochemical, coal-chemical and daily chemical industries [1].

Almost 60 years have passed since the first industrial application of synthetic Y zeolite in oil cracking, which led to a technical revolution in the refinery industry. So far, the Structure Committee of the International Zeolite Association (IZA) has certified >260 types of zeolite structures, of which nearly 20 have achieved industrial application, leading to a series of milestone technological revolutions in energy, chemical and environmental fields, as well as other fields, in the past few decades.

**Figure 1.**
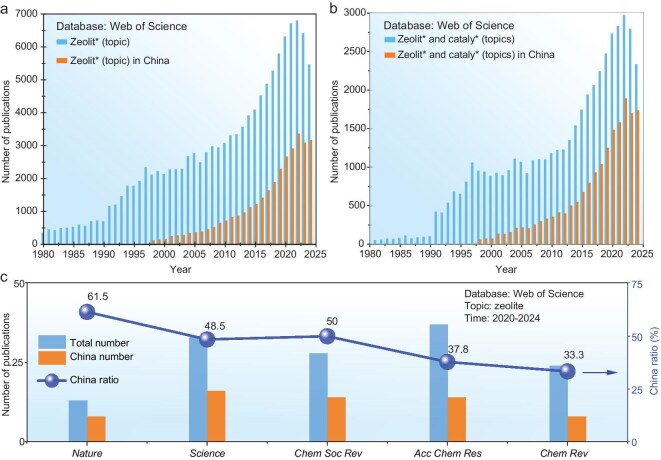
Number of publications related to (a) zeolite and (b) zeolite catalysis in China and in the world. (c) Publications of significant breakthroughs in zeolites by Chinese scholars and others.

Since the 1950s, a large number of Chinese researchers have devoted themselves to the field of zeolite research. A series of pioneering contributions to the scientific exploration and technological application of zeolite materials have been achieved, thereby establishing a robust foundation for the rapid growth of China’s petrochemical industry and related sectors. For example, the Y-7 low-cost semi-synthetic zeolite catalyst, developed under the direction of Prof. Enze Min in the 1970s, has allowed Chinese refining catalysts to compete with the world’s advanced standards, marking a leap forward in the field. He then spearheaded the development of residual oil catalytic cracking catalysts, along with their key active ingredients such as ultra-stable Y-type molecular sieves and rare-earth Y-type molecular sieves, to address the evolving needs of the Chinese refining industry and the upgrading of petroleum products [2]. Prof. Ruren Xu pioneered hydrothermal synthesis chemistry and was the first internationally to propose design and targeted synthesis pathways for zeolites with specific structures, thereby advancing the frontiers of molecular engineering for functional materials within China and globally [3]. Prof. Mingyuan He has also contributed to the development of zeolite research by integrating scientific insights with technological applications, developing innovative methods for the synthesis and modification of zeolites, and successfully fabricated various refining catalysts. His work has been instrumental in addressing key technological challenges in China, such as heavy oil cracking, enhanced octane ratings for catalytic cracking gasoline and the production of new standard gasoline [4]. However, in the domain of fundamental research, the Chinese zeolite research achievements were comparatively limited at that time. A paucity of papers published in international journals can illustrate this situation (Fig. 1a). The National Natural Science Foundation of China (NSFC) was established in 1986, with the approval of the State Council of China. The primary objective of the NSFC was to foster the advancement of fundamental research within China, thereby augmenting the nation’s scientific and technological innovation capacities. As the main funding source for basic research in China, the NSFC has contributed to the enhancement of China’s innovative capability and social and economic development by supporting basic research, talent training and research infrastructure while equally emphasizing international cooperation and interdisciplinary research.

In recent decades, global research on zeolites has undergone a significant resurgence, driven by advancements in synthetic chemistry, the development of characterization techniques and theoretical calculations, and the integration of diverse disciplinary fields. Concurrently, the number of scientific research publications has gone through rapid expansion. Over the past decade, China has increased its financial investment in the field of scientific research, with a significant number of zeolite-related projects having been approved by the NSFC. The scope of the project is broad, encompassing a wide range of project funding from the NSFC. It is evident that the proportion is comparatively elevated for projects with substantial funding amounts, including the Science Fund for Creative Research Groups, the Major Program, the Major Research Plan and the Excellent Research Group Program (formerly known as the Basic Science Center Program). China has witnessed a considerable augmentation in the number of scientific publications pertaining to zeolites, coinciding with its sustained commitment to scientific research (Fig. 1a).

Since 2020, the number of papers by domestic scholars has accounted for almost half of international publications, especially in the field of zeolite catalysis (Fig. 1b). Moreover, Chinese scholars have achieved significant breakthroughs in pioneering research endeavors. For instance, over the past 5 years, Chinese scholars have accounted for 61.5% and 48.5% of the publications related to zeolites in the *Nature* and *Science* journals, respectively (Fig. 1c). In other comprehensive review journals with high influence in the field of chemistry, such as *Chemical Society Reviews, Accounts of Chemical Research* and *Chemical Reviews*, the number of publications by Chinese scholars also reached 50%, 37.8% and 33.3%, respectively. In the field of zeolites, Chinese scientists have transitioned from a position of research followers to a leading role on the global stage. Some new concepts proposed by Chinese scholars have gradually been recognized and utilized by international peers, such as ‘single-atom catalysis’ proposed by Prof. Tao Zhang [5] and ‘confined catalysis’ proposed by Prof. Xinhe Bao [6]. In addition to the aforementioned achievements in fundamental research areas, several breakthroughs have been achieved in the field of industrial applications. For example, the world’s first industrial coal-to-olefins plant with an annual olefin-production capacity of 1 million tons, using the ‘Third-Generation Methanol-to-Olefins (DMTO-III) Technology’ led by Prof. Zhongmin Liu in the Dalian Institute of Chemical Physics (DICP), Chinese Academy of Sciences, was successfully put into operation in 2023 [7].

According to statistics and projections from Global Market Insights, the global annual demand for zeolite was ∼2 million tons in 2023, with China accounting for ∼30% of the total, generating an economic benefit of ∼$5 billion. The annual growth rate is expected to remain at 4.5% until 2029. The accelerated advancement of Chinese zeolite science and technology has been significantly influenced by the substantial support provided by the Chinese government, including the NSFC. This paper will provide a comprehensive review of the funding projects from the NSFC in the field of zeolite research. The review will consist of an overview of the funded projects and a corresponding spotlight on important developments and future prospects.

## OVERVIEW OF FUNDING PROJECTS RELATED TO ZEOLITE IN CHEMISTRY AT THE NSFC

The Department of Chemical Sciences at the NSFC comprises nine subjects. Synthetic Chemistry (**B01**, subject code) represents the primary undertaking within the domain of chemistry. Catalysis and Surface/Interface Chemistry (**B02**), Chemical Theory and Mechanism (**B03**) and Chemical Measurement Science (**B04**) constitute the theoretical underpinnings and technological modalities essential for fundamental research. The following chemical interdisciplines are of particular relevance in this context: Material Chemistry (**B05**), Environmental Chemistry (**B06**), Chemical Biology (**B07**) and Energy Chemistry (**B09**). These interdisciplines are concerned with the chemical aspects of material, energy, environmental and biological issues, and aim to provide solutions to some of the most pressing problems of our time. Chemical Engineering and Industrial Chemistry (**B08**) is an application-driven subject that aims to provide a scientific foundation and technological support for national major demands [8]. The Department of Chemical Sciences is dedicated to the enhancement of the overall quality and international status of fundamental research in the fields of chemistry and chemical engineering in China. The primary domains of funding will, in a secondary manner, mirror the trajectory of our fundamental and applied basic research in chemistry and chemical engineering. Zeolite—a pivotal material that serves as a nexus between chemistry and chemical engineering—will be utilized as a case study to analyse its funding situation and effectiveness in the field of chemistry over the past 5 years.

**Figure 2.**
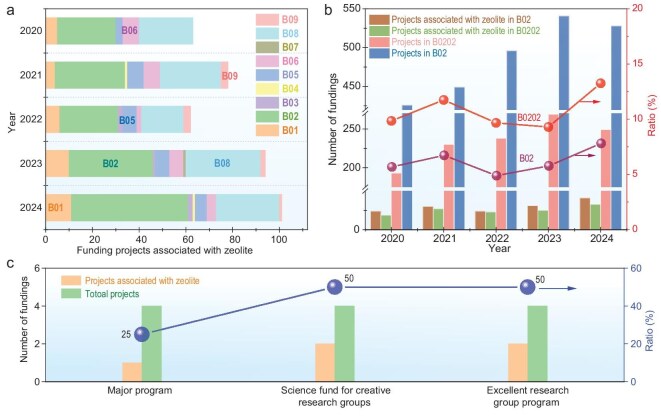
(a) Number of funding projects related to zeolite in the Department of Chemical Sciences over the past 5 years. (b) Number of total funding projects and those related to zeolite in B0202 and B02. (c) Number of funding projects with higher amounts and those related to zeolite in B02 over the past 5 years.

The subject of zeolite materials is broad and encompasses numerous aspects, including synthesis, characterization and application. Consequently, the relevant funding projects pertaining to zeolite in the Department of Chemical Sciences at the NSFC encompass multiple subject codes. The number of corresponding projects over the past 5 years is illustrated in Fig. 2a. It is evident that the funding projects pertaining to zeolite are distributed across a range of five to eight academic disciplines, with a notable concentration observed in the B02 and B08 disciplines. Furthermore, there has been a gradual yet consistent increase in the number of funding projects over the past 5 years, with the exception of 2021. Notably, in 2024, the majority of these projects were concentrated within the B02 discipline. With a focus on the B02 discipline, which contains five secondary codes, including Basic Theory and Characterization (B0201), Catalytic Chemistry (B0202), Surface Chemistry (B0203), Colloid and Interface Chemistry (B0204) and Electrochemistry (B0205), the number of funding projects in the secondary code of B0202 constitutes ∼50% of the total B02 discipline and the proportion has remained stable over the past 5 years (Fig. 2b). The funding proportions of zeolite-related projects in B0202 and the total funding projects in B0202 and B02 have remained stable and increased marginally over the past 5 years, within the ranges of 5%–8% and 10%–13%, respectively. It is noteworthy that the proportions of zeolite-related projects funded with higher funding amounts, such as the Major Program, Science Fund for Creative Research Groups and Excellent Research Group Program (formerly known as the Basic Science Center Program) within the B02 discipline, are very high, reaching 25%, 50% and 50%, respectively (Fig. 2c). It is evident that the objective of the Excellent Research Group Program is to unit domestic scientific research resources that are deemed advantageous, with a view to targeting international science frontiers, advancing deployment, leveraging the advantages and characteristics of the science funding system and relying on high-level academic leaders. The program also seeks to attract and assemble outstanding Science and Technology (S&T) talents, promoting in-depth cross-discipline integration and supporting scientific personnel to conduct research and exploration in a relatively long-term and stable manner, with the aim of bringing about important breakthroughs [8]. Consequently, the high proportion of zeolite-related projects in the Excellent Research Group Program within the B02 discipline indicates that research directions related to zeolite play a significant role in the B02 discipline.

**Figure 3.**
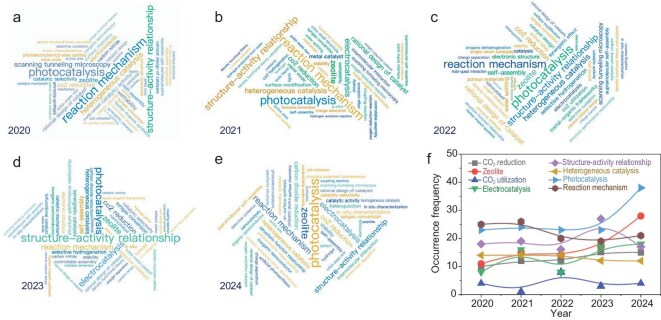
Cloud graphs of the main keywords in the funding projects in B02 in (a) 2020, (b) 2021, (c) 2022, (d) 2023 and (e) 2024 and (f) the corresponding occurrence frequency of the top eight keywords that appeared in the cloud graphs.

Given the centralization of zeolite-related funding projects within the B02 discipline, this paper will analyse the current research hot topics of Chinese scholars in this discipline. The top 50 keywords from the funding projects in B02 over the past 5 years are indexed and displayed in Fig. 3a–e. It is evident that the most frequently indexed keywords over the past 5 years encompass a wide range of subjects, including CO_2_ reduction, CO_2_ utilization, zeolite, electrocatalysis, heterogeneous catalysis, structure–activity relationship, photocatalysis and reaction mechanism. As demonstrated in Fig. 3f, over the course of the 2020–2023 period, the most frequently occurring indexed keywords were reaction mechanism, photocatalysis and structure–activity relationship. The high funding directions of reaction mechanism and structure–activity relationship clearly indicate that the B02 discipline emphasizes fundamental scientific research, aligning with its original intention to investigate catalytic processes and the structure and properties of surfaces/interfaces, and to reveal the physical and chemical principles underlying catalysis and surfaces/interfaces. From 2022 onwards, there has been a slight fluctuation in research interests in these two areas, yet they remain dominant. Photocatalysis has also emerged as one of the most indexed keywords, becoming the top indexed keyword in 2024. This may be associated with the energy strategy, as the advancement of sustainable and clean energy represents a significant method of attaining carbon-peaking and carbon-neutrality objectives. Furthermore, electrocatalysis has been identified as a significant research area, with a notable increase in research interest observed between 2022 and 2024 (Fig. 3f). For other keywords, such as CO_2_ reduction/utilization and heterogeneous catalysis, research interests have either remained stable or have experienced a slight increase over the past 5 years. It is noteworthy that interest in zeolite research is increasing. This may be indicative of the rapid advancements being made in the field of scientific research tools and computational chemistry, which provide a robust foundation for the exploration of scientific inquiries within the zeolite domain.

**Figure 4.**
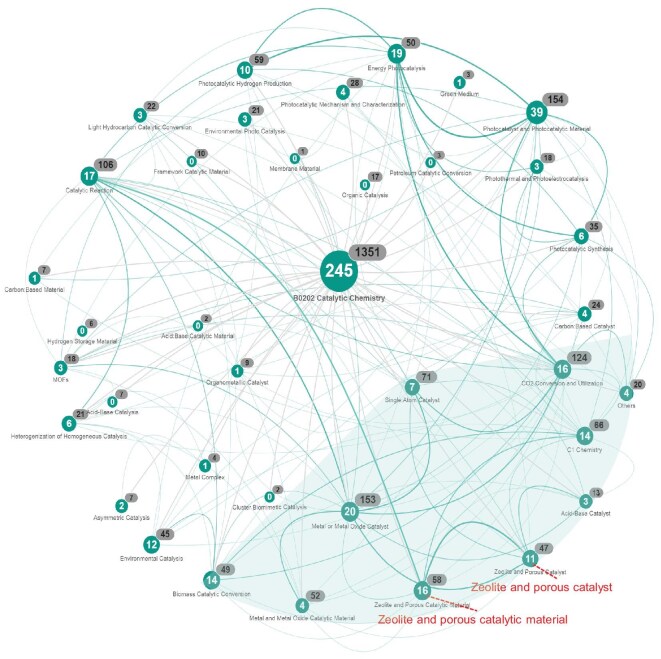
Interdisciplinary intensity among the funding projects for B0202 code in B02 discipline.

Zeolite, as a rapidly developing research direction in the B02 discipline, especially in the B0202 subdiscipline, has been identified as a key area of interest. The interdisciplinary intensity among the funding projects for 36 research directions in the B0202 code in 2024 is compared to obtain more information on their correlation with other research directions (Fig. 4). The white and black numbers in each research direction represent the application and funding numbers, respectively. It is evident that a significant proportion of the allocated funding number, amounting to 27 (Zeolite and Porous Catalytic Material & Zeolite and Porous Catalyst), has been designated for zeolite-related projects within the B0202 subdiscipline. This is only second to the projects pertaining to Photocatalyst and Photocatalytic Material, then followed by Metal or Metal Oxide Catalyst (20), Energy Photocatalysis (19), Catalytic Reaction (17), CO_2_ Conversion and Utilization (16), C1 Chemistry (14), Biomass Catalytic Conversion (14) and Environmental Catalysis (12), in that order. Evidently, there is a robust correlation between zeolite and porous catalytic material, suggesting that zeolite materials are predominantly employed in the field of catalysis. This finding is in close agreement with the established correlations with a range of areas, including Catalytic Reaction, Metal or Metal Oxide Catalyst, C1 Chemistry, Acid–Base Catalyst, CO_2_ Conversion and Utilization, Single-Atom Catalyst, Biomass Catalytic Conversion and more. Additionally, Zeolite and Porous Catalyst exhibit strong correlations with Metal or Metal Oxide Catalyst and Single-Atom Catalyst. This finding aligns with the recent focus of international research in the field, including metal-encapsulated catalysts, single-atom catalysts and oxide–zeolite (OXZEO) catalysts. It is noteworthy that, in comparison with zeolite, the correlations between CO_2_ Conversion and Utilization and Energy Photocatalysis, as well as Photocatalyst and Photocatalytic Material, are considerably more pronounced. This finding suggests a transition in the research focus from thermal catalysis to photocatalysis in the field of CO_2_ Conversion and Utilization.

Overall, the distribution and types of funding projects related to zeolites in chemistry at the NSFC over the past 5 years align well with its primary objective of strengthening both basic and applied basic research. The considerable proportion of these projects within larger ones, such as the Excellent Research Group Program, indicates that zeolite research has become one of the most cutting-edge fields. In the following section, the focus will be on the significant research advancements made by Chinese scholars in the field of zeolites that were supported by a grant from the NSFC.

## SPOTLIGHTS ON IMPORTANT PROGRESS IN THE ZEOLITE FIELD UNDER A GRANT FROM THE NSFC

### Zeolite synthesis

Over the past decade, under a grant of the NSFC, Chinese scholars have achieved a series of groundbreaking advancements in the design and synthesis of novel zeolites and functional applications. Wu and Xu *et al.* developed a series of novel zeolites, designated ECNU-n, through a combination of bottom-up and top-down strategies [9–11]. It is acknowledged that there are two members in ECNU-n, namely ECNU-16 and ECNU-21, which have been formally recognized and assigned three-letter structure codes, EOS and EWO, respectively, by the International Zeolite Association Structure Commission (IZA-SC). The creation of a series of new zeolites, SCM-14 and SCM-15, with structural codes of SOR and SOV, respectively, was achieved by Yang’s group. This was based on material genome engineering technology and a high-throughput system of zeolite synthesis and characterization. Utilizing the SCM-14 zeolite as the active component, a novel generation of highly selective butene isomerization catalysts was developed and industrial production was successfully achieved [12].

**Figure 5.**
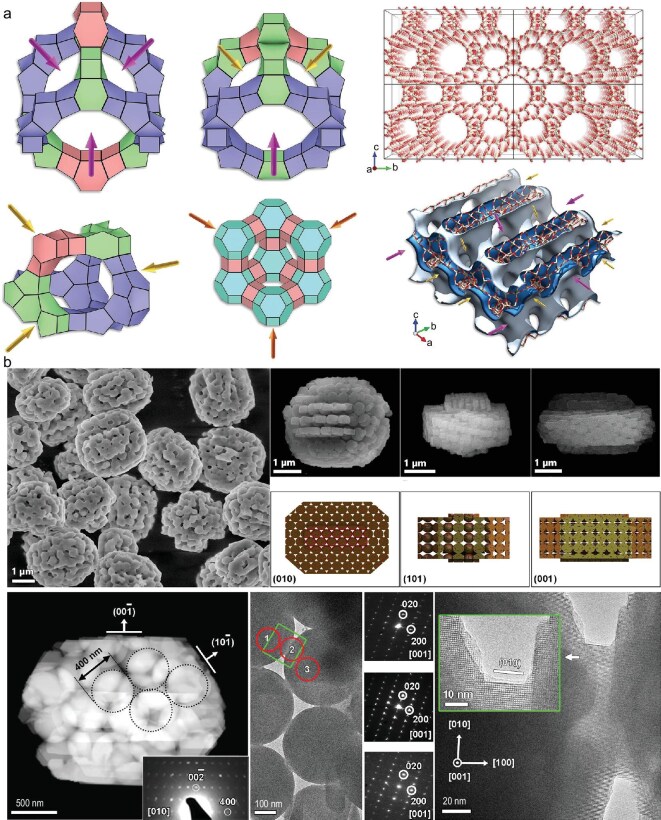
(a) Three supercages and channel system in ZEO-1 and FAU [13]. (b) TEM characterization of hierarchical macro–meso–microporous ZSM-5 single crystals [18].

Diffusion and mass-transfer performance are pivotal factors in determining catalytic efficiency. Zeolites are characterized by micropores of a size that imposes limitations on diffusion in a series of significant catalytic reaction processes, especially those involving macromolecular catalytic reactions. For a period exceeding eight decades, research into zeolite synthesis has been characterized by a concerted effort to identify 3D stable extra-large-pore zeolites, with the objective of enhancing diffusion and mass-transfer performance. This endeavor has consistently represented a significant challenge within this field. Yu’s group reports the synthesis of the first 3D stable extra-large-pore aluminosilicate zeolite ZEO-1, representing a major breakthrough in the field of zeolite science (Fig. 5a) [13]. A recently discovered mechanism of 1D-to-3D topotactic condensation has been described as a ‘click reaction’ in the context of zeolite synthesis. This discovery has enabled the synthesis of 3D stable extra-large-pore zeolites ZEO-3, ZEO-4 and ZEO-5, which continuously expand the limits of stable zeolites with regard to pore size [14,15]. Hierarchically porous zeolite single crystals with ordered and tunable pores could also offer the ideal solution to minimize the diffusion limitations and improve the catalyst efficiency [16]. Nevertheless, the synthesis of these compounds remains a highly challenging process. Chen and Su’s group have proposed a novel concept in the field of pore science and engineering. This development follows extensive research into the hierarchical structures of natural efficient material transport and energy conversion, and represents an expansion of the single-pore 1.0 system to the hierarchical-pore 2.0 system [17]. For instance, the synthesis of zeolite single crystals (e.g. ZSM-5, Beta, TS-1 and SAPO-34) with a fully interconnected and ordered intracrystalline macro–meso–microporous hierarchy was achieved, thereby offering accelerated mass transport and increased catalytic performances (Fig. 5b) [18,19]. In a recent report, the targeted synthesis of hierarchical porous Murray zeolites following the generalized Murray’s Law was described. These zeolites exhibit accelerated mass transfer and improved catalytic performance [20].

The formation process of zeolites has long been regarded as a ‘black box’ and the function-oriented and targeted design and synthesis of molecular sieve materials is a cutting-edge and exceedingly challenging issue in contemporary synthetic chemistry. The research conducted by Yu’s group has provided a comprehensive elucidation of the crystallization mechanism by which hydroxyl radicals accelerate the nucleation of zeolites [21]. This significant finding offers a valuable insight into the crystallization mechanisms of zeolite, thereby contributing to the advancement of the field. Recently, Wu and colleagues employed time-resolved 3D electron diffraction to reveal the topotactic reactions and transformations at the atomic scale. This process was studied from the starting phase, through various reaction intermediates at distinct time points, to the final product [11]. Very recently, Dai and Hou *et al*. clarified the intricate cooperative and competitive interactions of dual-template agents during CHA zeolite crystallization by means of a novel operando 2D high-temperature and high-pressure solid-state nuclear magnetic resonance (ssNMR) spectroscopy [22]. This innovative approach has the potential to significantly advance our understanding of molecular dynamics, crystallization mechanisms and synthesis methods for zeolites.

### Novel characterization techniques for zeolite materials

The bulk structure of zeolites can be determined through diffraction techniques, while their numerous non-periodic local structures, such as surfaces, interfaces, intergrowth of polymorphs, structural defects and guest molecules, cannot be directly resolved from diffraction data. Transmission electron microscopy (TEM) is the most effective method for characterizing the local structures of crystalline materials. However, due to the high sensitivity of zeolite structures, especially those with low Si/Al ratios, to electron beam irradiation, low-dose electron microscopy techniques are required to obtain reliable structural insights.

**Figure 6.**
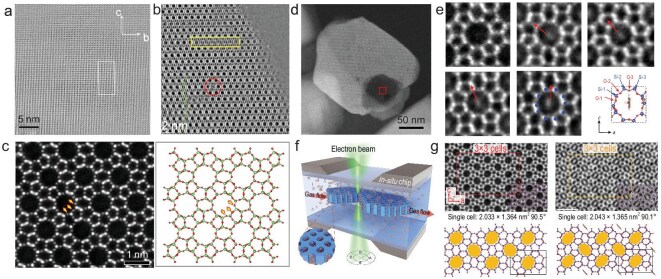
(a) Low-dose high-resolution transmission electron microscopy (HRTEM) image of a 0.8-nm-thick sodalite precursor (RUB-15) nanosheet, demonstrating its high crystallinity [23]. (b) iDPC-STEM image of zeolite EMM-17, revealing stacking faults and various types of point defects, as indicated by the label [24]. (c) Electron ptychography reconstructed image of MFI zeolite, along with the projected structural model. The achieved resolution of ∼0.85 Å enables clear identification of all framework atoms, including oxygen atoms, which are marked with green arrows. Imaging single aromatic molecules confined in the straight channels of ZSM-5 zeolite [25]. (d) Annular dark-field scanning transmission electron microscopy (ADF-STEM) image of an etched ZSM-5 crystal with thin areas for imaging confined molecules. (e) iDPC-STEM image of straight channel filled by para-xylene molecules in different orientations [27]. (f, g) *In situ* observation of sub-cell topological flexibility in zeolite channels during benzene adsorption/desorption process [28].

Han’s group has utilized low-dose high-resolution TEM integrating differential phase contrast scanning TEM (iDPC-STEM) to demonstrate the crystalline nature of a 0.8-nm-thick sodalite precursor (RUB-15) nanosheet (Fig. 6a) [23], revealing abundant point defects in EMM-17 (Fig. 6b) [24] and achieving the groundbreaking direct imaging of framework oxygen atoms in ZSM-5 (Fig. 6c) [25]. In a particularly notable study on Mo/ZSM-5, the researchers leveraged the specific interactions between Mo atoms within the micropores and Al sites in the zeolite framework to determine the preferential locations of Al atoms by precisely imaging the positions of Mo atoms [26]. The ability to distinguish between Si and Al atoms within the zeolite framework has proven to be a persistent challenge and the present study proposes a novel approach to address this issue.

The host–guest van der Waals interactions in nanoscale channels of zeolite are known to determine various physical and chemical behaviors of molecules confined within them, such as adsorption, transport and catalysis. However, it is difficult to investigate these interactions at the single-molecule level by using current methods. Wei and Chen *et al*. employed the low-dose electron microscopy technique to directly observe the dynamic behavior of guest molecules within sub-nanometer pores. They proposed a high-temperature imaging method for confined small molecules in zeolites, achieving the first microscopic observation of the confined state of aromatic hydrocarbon molecules (Fig. 6d and e) [27]. Furthermore, the transport process of molecules entering and exiting zeolite pores was observed, leading to the discovery that zeolites possess intrinsic topological flexibility (Fig. 6f and g). This elucidates the microscopic mechanism by which large molecules can be accommodated within small zeolite pores, resulting in substantial local deformation, thereby enhancing the comprehension of shape-selective catalysis [28,29]. Furthermore, the group successfully achieved the atomic-level imaging of strongly adsorbed small molecules (e.g. pyridine and thiophene), thereby unveiling spatial heterogeneity and specificity in the adsorption behavior of guest molecules. Within local channels, these molecules are influenced by varying numbers of acidic sites, making desorption extremely difficult at high temperatures [30,31]. These works have pioneered a new field of studying the dynamic behavior of confined small molecules and host–guest interactions at the atomic scale, thus laying a solid foundation for future *in situ* and real-time studies of mass diffusion and transformation processes.

**Figure 7.**
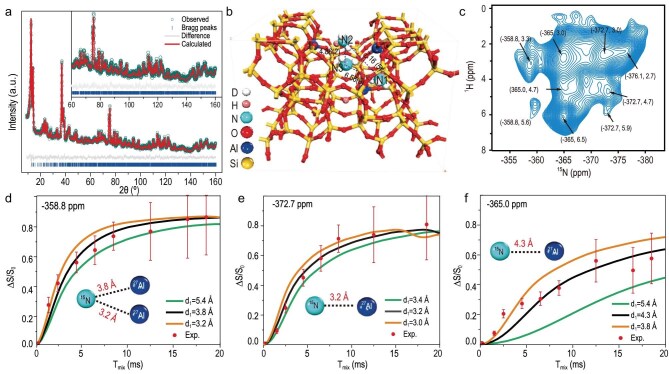
(a) Rietveld refinement profiles of NPD data [wavelength, l = 2.43953(2) Å; weighted profile residual, Rwp = 1.77%; expected profile residual, Rexp = 1.54%; GoF = 1.15]. Zeolite structures with the determined ND_3_ sites (N1, N2 and N3) with the bond-length information (in angstroms) retrieved by means of NPD–rietveld refinement. (b) Refined periodic structure. (c) 2D ^15^N–^1^H HETCOR MAS SSNMR spectra of ^15^NH_3_ adsorbed on H-ZSM-5. ^15^N–^27^Al S-RESPDOR built-up data and simulated curves by plotting the signal fraction DS/S_0_ = (S_0_ – S′)/S_0_ (S_0_ and S′ represent the signal intensity with and without dipolar dephasing, respectively) as a function of the recoupling time *t*, for ^15^N chemical shift at (d) –358.8, (e) –372.7 and (f) –365.0 ppm. d1 represents the internuclear distance of the ^15^N–^27^Al spin pair [32].

The distribution of substitutional aluminum (Al) atoms in zeolites has been demonstrated to affect molecular adsorbate geometry, catalytic activity, and shape and size selectivity. The accurate determination of Al positions has proven to be a formidable challenge. In a recent study, Deng *et al*. collaborated with foreign scholars to precisely locate ‘single Al’ and ‘Al pairs’ in a commercial H-ZSM-5 zeolite. This was achieved by combining neutron powder diffraction (NPD) with molecule adsorption ssNMR techniques [32]. According to the ^15^ND_3_ adsorption NRSXRD (Fig. 7a and b) and ^15^NH_3_ adsorption ssNMR (Fig. 7c–f), the precise atomic locations of Al can be obtained, namely the T8, T6 and T4 sites. That is to say, T8 is located at the straight channel, T6 in the cross-channel voids and T4 in the sinusoidal channel. Very recently, Hou’s group developed a novel strategy by employing divalent cation titration with varying cation sizes, in combination with advanced quantitative ^1^H NMR and ^1^H–^1^H homonuclear correlation techniques, to accurately identify and classify three distinct types of Al pairs [33]. It is evident that the Al pairs are aligned along both six-membered rings (6-MRs) and 10-membered rings (10-MRs), in addition to being found in disparate channels. These methodologies offer a highly effective approach to the investigation of Al, its positioning and its proximity, which provides valuable insights into their structural and catalytic properties.

### Adsorption and diffusion behavior of guest molecules within zeolite pores

**Figure 8.**
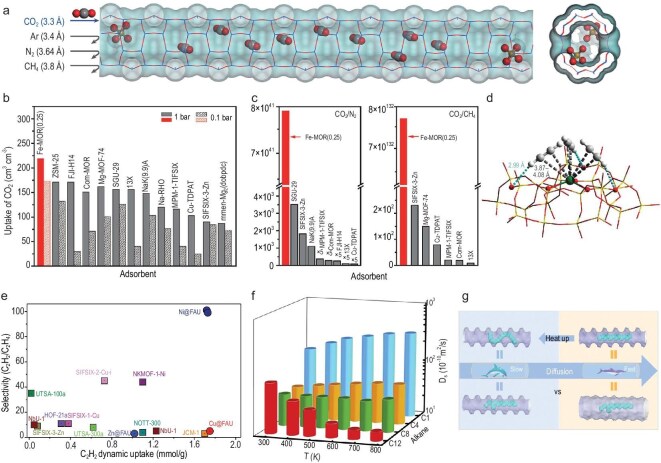
(a) Side view and top view of precisely narrowed microchannels (kinetic diameter: 3.3–3.4 Å) by occupying isolated tetrahedral Fe species inside the 12-MR MOR microchannel. Blue indicates Si or Al, red indicates O, light brown indicates Fe and gray indicates C. (b) Comparison of volumetric CO_2_ uptakes at 298 K. (c) Comparison of CO_2_/N_2_ and CO_2_/CH_4_ IAST selectivities at 1 bar and 298 K, respectively [34]. (d) Formation of meta-stable [Ni(C_2_H_2_)_3_] complexes within the supercage of faujasite. (e) Plot of C_2_H_2_/C_2_H_4_ dynamic selectivity against C_2_H_2_ dynamic uptake under ambient conditions with state-of-the-art sorbent materials [35]. (f) Self-diffusion coefficients of methane (C1), *n*-butane (C4), *n*-octane (C8) and *n*-dodecane (C12) in MFI zeolite at different temperatures. (g) Scheme of ‘thermal resistance effect’ (TRE) for C12 molecule diffused inside zeolite channels at low and high temperatures [36].

The separation of gas molecules by using physisorbents can present certain challenges, due to the inherent trade-off between capacity and selectivity. The utilization of ordered microporous materials as potential candidates for physisorbents is predicated on their molecular sieving ability, which confers excellent selectivity. Zeolites, for instance, are low-cost and thermally and hydrothermally stable, and have been applied on large scales as catalysts and sorbents. Various approaches have been proposed to improve the sorption performance of zeolites by manipulating their topology, morphology and porosity. Nevertheless, the precise control of the zeolite pore aperture within the kinetic diameters of gas molecules is challenging. Wang *et al*. developed a template-free hydrothermal synthesis method of mordenite zeolite (MOR) in which partial silicon atoms were replaced by iron atoms (Fig. 8a) [34]. The Fe–MOR monoliths, having been prepared in advance, were found to be suitable for immediate industrial application, achieving unprecedented levels of volumetric CO_2_ uptake (Fig. 8b). In lieu of forming a powder that necessitates additional shaping, this mechanically stable material self-assembled into monoliths. Iron atoms bound in tetrahedral zeolite sites narrowed the channels and enabled the size-exclusion separation of carbon dioxide (CO_2_) over nitrogen (N_2_) and methane (CH_4_) (Fig. 8c).

Li *et al*. developed a straightforward strategy to confine isolated transition metal sites (M = Ni, Cu, Zn) in the 6-MRs of faujasite, denoted as M@FAU [35]. Under ambient conditions, Ni@FAU demonstrated the remarkable adsorption of alkynes and efficient separation of alkyne/olefin mixtures, exhibiting state-of-the-art performance. The underlying separation mechanism of Ni@FAU was fully elucidated by employing a combination of *in situ* NPD, inelastic neutron scattering and density functional theory calculations. These complementary techniques revealed that the confined Ni(II) sites within the 6-MRs enabled the chemoselective and fully reversible binding to alkynes via the formation of meta-stable [Ni(alkyne)_3_] complexes (Fig. 8d). The open Ni(II) sites in Ni@FAU have been demonstrated to impede olefin adsorption through the expeditious displacement of adsorbed olefins by alkynes under dynamic conditions, thereby yielding unparalleled separation selectivity of acetylene/ethylene (Fig. 8e).

The adsorption and diffusion behavior within zeolitic pores plays a pivotal role in determining the efficiency of reactant molecules accessing the active sites, thereby fundamentally influencing catalytic activity and selectivity. The adsorption and diffusion behavior of zeolites is governed by their well-defined pore sizes, geometric structures, tunable surface chemistry and high-density active centers, which can be easily quantified at room temperature. It is challenging to ascertain the diffusion behavior of reactant or product molecules in the zeolite channels when the reaction temperature is elevated. It is a well-established fact that diffusion is generally faster at higher temperatures. However, Zheng *et al.* discovered a counterintuitive phenomenon termed the thermal resistance effect, by which the diffusion of long-chain hydrocarbons (e.g. *n*-dodecane, C_12_) in confined zeolite channels (e.g. MFI, MTW) slows with increasing temperature, contrary to conventional Arrhenius behavior (Fig. 8f) [36]. The study’s key findings indicate that elevated temperatures induce severe structural deformation in linear-chain molecules, causing them to adopt branched-chain-like configurations (Fig. 8g). This distortion has been shown to increase steric hindrance, thereby raising diffusion resistance and reducing self-diffusion coefficients (e.g. C_12_’s Self-diffusion coefficients (Ds) drops from 0.76 × 10^−8^ m^2^/s at 298 K to 0.15 × 10^−8^ m^2^/s at 773 K).

### Zeolite catalysis

In recent years, significant investment in scientific research has been directed towards the field of zeolite catalysis, leading to noteworthy advancements by Chinese scholars. The nation has emerged as a prominent figure in this research domain, eclipsing its previous status as a follower and attaining a leadership position.

The ‘single-atom catalysis’ proposed by Zhang *et al*. [5,37] has received intensive attention internationally in the heterogeneous catalysis field. Recently, Wang *et al*. developed a strategy for designing stable single-atom catalysts by harnessing a second metal to anchor the noble metal atom inside zeolite channels. This RhIn4@silicalite-1catalyst demonstrates exceptional stability against coke formation for 5500 h in continuous pure propane dehydrogenation with 99% propylene selectivity and propane conversions close to the thermodynamic equilibrium value at 550°C [38]. Very recently, Huang *et al*. presented zeolite-confined Cu single-atom clusters that exhibited remarkable activity in an electrochemical CO reduction reaction [39]. Typically, the Cu-based membrane electrode assembly can stably catalyse CO to acetate at an industrial current density of 1 A cm^−2^ at 2.7 V (Faraday efficiency 61 ± 5%) beyond 1000 h at atmospheric pressure.

**Figure 9.**
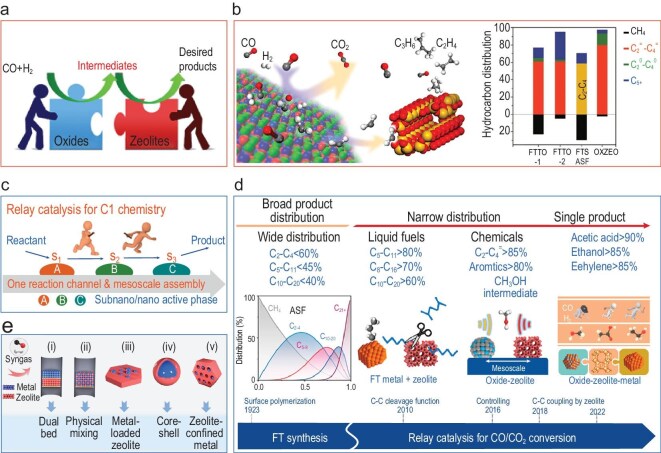
(a) Cooperation of the two functions within the framework of OXZEO catalyst design concept [40]. (b) OXZEO bifunctional catalyst concept for syngas conversion to light olefins and the hydrocarbon distribution in comparison with the ASF distribution in conventional FTS and FTTO-1 over an Fe-based catalyst and FTTO-2 over a Co-based catalyst [6,40]. (c) Schematic model of relay-catalysis systems. (d) Development of relay-catalysis systems for CO/CO_2_ hydrogenation. (e) Representative manners of assembly of relay catalysts composed of metal nanoparticles and zeolites [44].

Bao *et al*. designed a bifunctional catalyst based on a metal oxide and zeolite, namely the OXZEO catalyst (Fig. 9a), instead of using metals or metal carbides as the Fisher-Tropsch Synthesis (FTS) catalyst [40]. In the OXZEO catalytic process, the activation of CO and H_2_, as well as the generation of active intermediates, was conducted over a partially reduced metal oxide surface. Subsequently, the intermediates underwent desorption into the gas phase and diffusion into the confined channels of the zeolites (or zeolite-type catalysts). There, C–C coupling occurred, forming hydrocarbon products. Consequently, direct syngas conversion is spatially separated into a tandem process with two active centers that respond to feedstock activation and hydrocarbon product formation (Fig. 9b) [6]. For instance, a bifunctional composite with a partially reduced ZnCrO*_x_* oxide and mesoporous SAPO-34 zeotype has been shown to enable direct syngas conversion to light olefins with selectivity as high as 80% at a CO conversion rate of 17%. The C_2_–C_4_ hydrocarbons, comprising olefins and paraffins, exhibited an increase of >90%. This outcome is notably higher than the Anderson-Schulz-Flory (ASF) model’s estimation of 58% and the yields achieved in the FTS-to-light-olefins (FTTO) process, which are 61% over Fe- or Co-based catalysts. Subsequently, it was determined that the ZnCrO*_x_*-SAPO-17 OXZEO catalyst possessed the capacity to enhance ethylene selectivity while concomitantly diminishing C_4+_ selectivity during the induction phase of syngas conversion [41]. ZnCrO*_x_*-GeAPO-18 catalysts have recently been developed and it has been demonstrated that these catalysts can overcome the activity–selectivity trade-off effect for the syngas-to-light olefins reaction [42]. It is possible to achieve simultaneous CO conversion and selectivity of >80% for light olefins. Consequently, an unprecedented light-olefin yield of 48% can be attained. The OXZEO concept has been recognized by numerous international peers and has led to the development of C1 chemistry, including syngas and CO_2_ conversion. It has been demonstrated that multiple reactions can be achieved through the utilization of synthesis gas conversion and CO_2_ conversion.

The superior product selectivity of OXZEO, which surpasses that of conventional FTS, has also garnered attention from industry. Bao’s team collaborated with the applied development team led by Liu at the DICP, with the objective of developing industrially scaled-up catalysts and technology for direct syngas conversion to light olefins (OXZEO-TO). Furthermore, a collaboration was initiated with the Yanchang Petroleum Group, resulting in the execution of the inaugural industrial pilot test, which yielded an annual output of 1000 t of light olefins in 2020. This endeavor served to substantiate the scientific principle and the viability of the process [40,43].

Wang *et al*. developed a relay-catalysis strategy to enable the controlled, efficient hydrogenation of carbon monoxide and carbon dioxide with C–C coupling [44]. The objective is to devise a single reaction channel with clearly defined intermediates and reaction steps, which are to be connected in such a manner as to guide the reaction in a way that is analogous to a relay race, with the end goal of achieving the desired product (Fig. 9c). The capacity of relay catalysis to disentangle intricate reaction networks comprising sequentially arranged steps renders it a promising candidate for achieving high selectivity in target products. In recent years, the Wang group has employed the relay-catalysis strategy for the selective hydrogenation of CO/CO_2_ to C_2+_ compounds, thereby achieving a selective breakdown of the ASF distribution (Fig. 9d). These systems are typically composed of a metal or metal oxide for CO/CO_2_/H_2_ activation and a zeolite for the selective formation of products. A range of integration methodologies have been developed for the construction of integrated systems (Fig. 9e). In comparison with integration modes (i) and (ii), mode (iii), i.e. the metal-loaded zeolite, has been shown to exhibit greater intimate contact between the two components and, as such, has been adopted in a number of studies.

**Figure 10.**
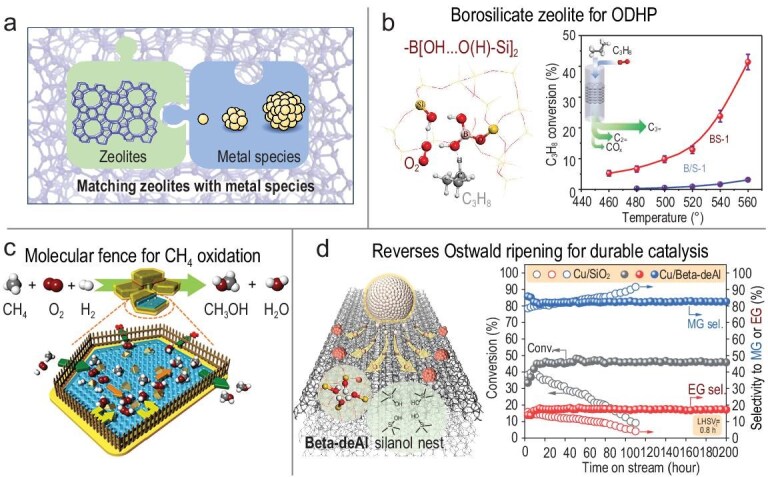
(a) Schematic model on metal–zeolite composite [45]. (b) Borosilicate zeolites and application in oxidative dehydrogenation of propane (ODHP) [38]. (c) Schematic model of molecular fence for CH_4_ oxidation [48]. (d) Reverse Ostwald ripening for durable copper nanoparticle catalysts in dimethyl oxalate hydrogenation reaction [50].

Metal–zeolite composite catalysts have also emerged as a promising frontier in catalysis, gaining significant attention from both academic and industrial communities. The combination of high-activity metal active sites with the shape selectivity and thermal stability of zeolites (Fig. 10a) results in excellent performance across many important reactions [45]. In the preceding half-decade, Xiao and colleagues have developed a series of metal–zeolites and demonstrated the remarkable catalytic activity of these in several significant chemical reactions [46–48]. For instance, they developed borosilicate zeolites featuring a unique –B[OH…O(H)–Si]_2_ structure, which exhibit high activity in the oxidative dehydrogenation of propane (Fig. 10b) [49]. Furthermore, a molecular-fence design concept was proposed, entailing the embedding of AuPd alloy nanoparticles within aluminosilicate zeolite, with subsequent modification of the external surface of the zeolite with organosilanes. This results in a zeolite nanoreactor with a high local concentration of H_2_O_2_, which considerably enhances the efficient oxidation of methane (Fig. 10c) [50]. In a recent study, the reverse-ripening phenomenon of Cu nanoparticles (NPs) on a dealuminated Beta zeolite support was demonstrated. It was observed that the Cu NPs tended to become smaller under the methanol vapor treatment (Fig. 10d). This feature enables the realization of an ideal catalyst with superior durability for the hydrogenation of Dimethyl Oxalate (DMO) that outperforms the classic Cu–silica-based catalysts [51]. Cao *et al*. constructed a novel Rh@MEL zeolite that exhibited >99% regioselectivity to *n*-butanal and >99% selectivity to aldehydes at a product-formation turnover frequency of 6500 h⁻¹ in the regioselective hydroformylation of propene. This performance surpasses that of all heterogeneous and most homogeneous catalysts developed to date [52,53].

In addition to the aforementioned achievements in the domain of fundamental research, noteworthy advancements have been made in the industrial application of zeolite catalysis. In 2020, the ‘Third-Generation Methanol-to-Olefins (DMTO-III) Technology’ led by Liu in the DICP, Chinese Academy of Sciences, passed the scientific and technological achievement appraisal organized by the China Petroleum and Chemical Industry Federation. In August 2023, the world’s first industrial coal-to-olefins plant, with an annual olefin-production capacity of 1 million tons, was successfully initiated (Ningxia Baofeng Energy Group Co., Ltd). This achievement was made possible by the application of the aforementioned technology. Furthermore, the industrialization of the ‘dimethyl ether-to-ethanol technology’ has been achieved, with the implementation of zeolite catalysis being led by Liu in collaboration with Shaanxi Yangchang Petroleum (Group) Co., Ltd. In 2022, a coal-based ethanol project with an annual production capacity of 500 000 tons was successfully initiated. It is noteworthy that the China Petroleum and Chemical Industry Federation awarded the Special Prize for Technological Invention in 2024 to ethanol technology.

The Sinopec Research Institute of Petroleum Processing Co., Ltd (RIPP) is an institution that utilizes zeolite catalysis in its research endeavors. RIPP has successfully developed bio-jet fuel production technology known as SRJET, utilizing used cooking oils as raw materials. This technology was implemented at Sinopec Zhenhai Refining & Chemical Company, which possesses an annual bio-jet fuel production capacity of 100 000 tons. The implementation resulted in China’s first bio-jet fuel product being utilized in commercial flights both domestically and internationally. It is noteworthy that the inaugural test flights of this bio-jet fuel product in China’s domestically manufactured large aircraft, designated C919, were conducted in 2024 and met with success. Based on MFI zeolite nanosheet, SINOPEC Shanghai Research Institute of Petrochemical Technology Co., Ltd (SRIPT) has developed a fully crystalline catalyst with high activity and stability for the vapor-phase alkylation of benzene with dilute ethylene to produce ethylbenzene. By adopting a new method, 50 tons of wastewater will be reduced for the production of every 1 ton of catalyst. In 2023, the catalyst was successfully implemented in an industrial unit with a capacity of 120 MTa. The technology was awarded the Gold Medal at the 50th International Exhibition of Inventions in Geneva. The SINOPEC Methanol-to-Olefins (SMTO) technology, together with second-generation OCC (olefins catalytic cracking) technology, is licensed to a gas chemical complex in Uzbekistan to enable the efficient use of natural gas and the production of high-value added polyolefins in demand both in Uzbekistan and abroad. The facility is expected to have a capacity of 1.34 million tons per year. Lately, SRIPT’s zeolite-based butene skeletal isomerization catalyst for producing isobutene has been exported to South Korea and delivered superior performance. Besides, using SCM-14 zeolite (Sinopec Composite Material No. 14) as the active component, a new catalyst for the double-bond isomerization of butene was developed and industrialized [12].

With the financial support of the NSFC, Chinese scholars have accomplished a number of pioneering achievements in the domains of zeolite catalysis, in both fundamental and applied research. A significant number of achievements have been made in the field of research, with researchers attaining the status of international leaders in their respective domains. These achievements align with the NSFC’s original intention of providing support for original and disruptive technological innovation, enhancing forward-looking, strategic and systematic planning in the domain of basic research, continuously increasing the effectiveness of scientific funding, optimizing the unique role of sustainable funds within the national innovation system and effectively supporting the realization of self-reliance and self-improvement in high-level conservation technology.

## CONCLUSION AND PROSPECTS

Scholars from China have made considerable progress in the field of zeolite research and have gradually become international leaders in this area under the support of the Chinese government, including the NSFC. At present, fundamental and applied fundamental research represent the primary orientations of national strategies for scientific development. Zeolites, characterized by both scientific significance and industrial applicability, serve as a typical prototype of the national strategy of integrating fundamental research and applied fundamental research. In the future, the NSFC is set to maintain its pivotal role in the development of zeolite research in China. With the aid of artificial intelligent, the NSFC is planning to construct a national zeolite database to guide research in the field of zeolite synthesis and catalysis. In order to facilitate the capacity of research groups to undertake intensive and in-depth studies, the NSFC seeks to enhance the continuity of scientific funding. This is intended to be achieved by means of the continuing funding of projects, such as the Science Fund for Creative Research Groups and the Major Program. In order to align with the national strategy of accelerating the commercialization of scientific findings, the NSFC will also explore the commercialization process of scientific and research findings in the field of zeolite research and further establish a platform for the efficient commercialization of scientific findings across multiple disciplines. Furthermore, the NSFC will consider the commercialization to be a significant criterion in the evaluation of the completion acceptance of funding projects. It is imperative that China maintains its position as a global leader in scientific research and this can only be achieved through effective international scientific communication. The NSFC is dedicated to the promotion of international scientific communication and intends to provide special funding for international scholars, with a particular emphasis on those from underdeveloped nations. Funding strategies are subject to constant optimization and scientific funding from the NSFC is expected to provide a solid scientific foundation and technological support for the development of new quality productive forces.
